# Intravenous Ketamine for Depressed and Suicidal Adolescents in the Emergency Department: A Randomized Double-Blind Trial

**DOI:** 10.5811/westjem.53968

**Published:** 2026-07-28

**Authors:** Tatyana Vayngortin, Ekta Patel, Shamilka Seneviratne, Daniel Lowet, Christian D. Saavedra Chavez, Christine Chau, Kathryn Hollenbach, Fareed Saleh, John Kanegaye

**Affiliations:** * University of California, San Diego School of Medicine, Department of Pediatrics, Division of Emergency Medicine, La Jolla, California; † Rady Children’s Hospital San Diego, San Diego, California; ‡ University of California, San Diego School of Medicine, Department of Psychiatry, La Jolla, California; § New York-Presbyterian/Weill Cornell Medicine, Department of Psychiatry, New York, New York; || Stanford University, Department of Psychiatry, Stanford, California; # Rady Children’s Hospital, Department of Pharmacy, San Diego, California

## Abstract

**Introduction:**

Adolescents frequently seek care in the emergency department (ED) for behavioral health emergencies. Due to national shortages in staffing, limited opportunities to begin treatment on site in most centers and, thus, the frequent default to elusive inpatient psychiatric beds as a means of stabilization, adolescents may spend prolonged amounts of time boarding in the ED, often without treatment. Ketamine is a rapid-acting antidepressant with emerging evidence in adults, but data are limited on its effectiveness in suicidal youth. We aimed to evaluate the effectiveness of a single dose of intravenous (IV) ketamine on depression and suicidality scores compared to placebo in adolescents with treatment-resistant depression presenting to the ED with suicidal ideation.

**Methods:**

We conducted a two-arm randomized double-blind placebo controlled trial on a convenience sample of adolescents 12–17 years of age with treatment-resistant depression (trialed at least two antidepressants for at least four weeks each) who presented to the ED with suicidal ideation requiring psychiatric admission. Exclusion criteria included psychosis, substance abuse disorder, intoxication, developmental delay, aggressive behavior, and medical contraindications to ketamine. Subjects were randomized to receive a single dose of ketamine (0.2 mg/kg IV, maximum 35 mg) or saline placebo over two minutes. They completed the Beck Youth Inventory (BYI) and Suicidal Ideation Questionnaire (SIQ) at baseline and at 1 hour, 3 hours, 1 day, 3 days, and 7 days post-treatment. We compared groups with the Fisher exact and two-sided rank-sum tests. Our primary outcome measure was the proportion of subjects with a reduction of ≥ 50% on the BYI and SIQ within 3 hours after treatment.

**Results:**

The 29 subjects (14 placebo, 15 ketamine) did not differ significantly in demographic and clinical characteristics. Despite reductions in BYI and SIQ in both groups, the proportions achieving ≥ 50% reductions did not differ significantly at 1 hour (SIQ: placebo, 0% vs ketamine 14.3 % [*P* = .48]; BYI Anxiety Inventory: placebo, 0% vs ketamine 21.4% [*P* = .22]; BYI Depression Inventory: placebo, 0% vs ketamine 15.4% [*P* = .22]). Differences at 3 hours were less pronounced. Adverse effects were more frequent in the ketamine group, most notably signs of dissociation (7% vs 60%, *P* = .004), with none after the 1-hour observation period.

**Conclusion:**

The ED offers an opportunity to deliver rapid-acting antidepressant treatment for suicidal adolescents. Although low-dose ketamine was not associated with greater reductions in depression or suicidality than placebo, this study demonstrated feasibility and highlights the need for larger studies to determine optimal ketamine dosing, route of administration, and implementation strategies in the ED setting.

## INTRODUCTION

In the United States, an estimated 3.7 million children and adolescents (14.7%) experience depression.^1^ Adolescents with depression are more likely to face mental health challenges in adulthood, as well as have poor academic performance, higher risk of unemployment, and increased susceptibility to substance abuse. The severity of illness has been increasing over the past 20 years, leading to suicide being the second leading cause of death in the U.S. among individuals 10–24 years of age.^2^ Moreover, about one-fourth of adolescents report having considered committing suicide over the prior year.^3^ The national youth mental health crisis was further exacerbated by the COVID-19 pandemic, when adolescents faced school closures, isolation, fear of illness, and decreased access to care.^2^

The emergency department (ED) often serves as a recommended primary destination for patients facing a behavioral health emergency, leading to a significant increase in ED use.^4^ Burstein et al reported a doubling of ED visits among youth for suicidal ideation and suicide attempts from 2007–2015.^5^ Yard et al compared adolescent ED visits for suicide attempts before the pandemic to after the onset of the pandemic, and described a 31% increase in 2020 compared to 2019, and a 39% increase in 2021 compared to 2019, with a more pronounced increase among females.^6^ As EDs continue to receive a growing volume of behavioral health emergencies, there remains a critical need for timely, EDbased interventions for imminently suicidal adolescents. While multiple systemic factors can contribute to prolonged ED length of stay (“boarding”), a central challenge is the limited availability of rapid, targeted treatments for acute suicidality in the emergency setting. Current ED management of suicidal patients focuses on safety assessment, observation, and disposition planning, rather than targeted treatment of suicidal ideation. Because the ED is often the only unavoidable access point for youth, developing effective acute treatments has the potential to reduce symptom severity during the ED stay and improve the care trajectory for these patients.

Unfortunately, severe youth depression is often refractory to current first-line treatments, including psychotherapy and pharmacotherapy with selective serotonin reuptake inhibitors (SSRI). There are only two SSRIs approved by the U,S. Food and Drug Administration for depression in youth, and roughly 40% of adolescents with depression fail to respond to initial treatment.^7^ Even among those who respond to medications, onset of antidepressant effect often takes several weeks. Ketamine, a rapid-acting N-methyl-D-aspartate receptor antagonist, has emerged as a promising intervention for rapid reduction of depressive symptoms and suicidal ideation. Ketamine acts by increasing the activity of glutamate, an excitatory neurotransmitter, in the frontal cortex. Ketamine also increases brain-derived neurotrophic factor, which stimulates synaptogenesis.^8^ Numerous studies over the past 20 years have demonstrated that ketamine at subdissociative doses has rapid and sustained antidepressant and antisuicidal effects in adults with mood disorders.^9^–^13^

**Population Health Research Capsule**

What do we already know about this issue?

*ED boarding is common for suicidal adolescents with limited treatment; ketamine shows rapid antidepressant effects in adults, but pediatric data are limited*.

What was the research question?

*Does intravenous (IV) ketamine reduce depression and suicidality compared to placebo in suicidal adolescents with treatment resistant depression in the ED?*

What was the major finding of the study?

*A single dose of IV ketamine did not significantly decrease depression or suicidality compared to placebo within 3 hours in depressed adolescents in the ED*.

How does this improve population health?

*The ED offers an opportunity to deliver rapid-acting antidepressant treatment for suicidal adolescents, which could lower the strain on EDs and inpatient psychiatry units*.

There have been limited studies on ketamine use for depression in adolescents, but they did find ketamine to be safe and effective for reducing depression and suicidality.^14^–^23^ While there is extensive data on the safety and efficacy of ketamine for procedural sedation and analgesia in adult and pediatric EDs,^24^ there is limited research on its use for mental health indications in this setting. Although ketamine 0.2 mg/kg intravenous (IV) reduces symptoms of depression and suicidality in the adult ED setting,^25^–^28^ no data exist on the effectiveness of ketamine for acute depression and suicidal ideation in the pediatric ED setting. Therefore, we sought to evaluate the efficacy and safety of ketamine in adolescents with acute suicidal ideation. We hypothesized that among adolescents with treatment-resistant depression presenting to the ED with suicidal ideation, a significantly greater proportion would have reductions of ≥ 50% on both the Beck Youth Inventory Questionnaire (BYI) and the Suicidal Ideation Questionnaire (SIQ/SIQ Jr)—measures of depression and suicidality, respectively—within three hours after a single subdissociative dose of ketamine 0.2 mg/kg IV than after placebo.

## METHODS

### Study Design

We conducted a two-arm, prospective, randomized double-blinded, placebo-controlled trial comparing ketamine to normal saline placebo. The study followed the Consolidated Standards of Reporting Trials (CONSORT) guidelines and was approved by the institutional review board of the University of California, San Diego (UCSD) and Rady Children’s Hospital Research.^29^ The study was registered under clinicaltrials.gov ID NCT05217706 and was exempt from an investigational new drug application.

### Study Setting and Population

Rady Children’s Hospital San Diego, an academic medical center affiliated with UCSD, serves as the region’s sole tertiary children’s hospital. The 63-bed pediatric ED has approximately 100,000 annual visits, 15% of which are by adolescents and 4% with behavioral health complaints. All patients with behavioral health complaints undergo evaluation by an emergency physician and social worker. They also complete a Patient Health Questionnaire-9 (PHQ-9), which consists of nine items to measure the severity of depression at a given time point. Scores 5–9 indicate mild depression, 10–14 moderate depression, 15–19 moderately severe depression, and ≥ 20 indicates severe depression.

### Subject Enrollment

Adolescents 12–17 years of age were eligible to participate in the study if they presented to the ED with a chief complaint of suicidal ideation or suicide attempt, had treatment-resistant depression, and required inpatient psychiatric care on the basis of involuntary hold or parental voluntary admission. Treatment-resistant depression was defined as having trialed two antidepressant medications for at least four weeks each without improvement.^30^ Exclusion criteria included the following: non-English speaking; history of a primary psychotic disorder; current psychotic episode; no parent/guardian to provide consent; a history of substance use disorder in the prior six months; acute intoxication; homicidal ideation or aggressive behavior; current use of medication that could interact with ketamine due to its effect on N-methyl-D-aspartate receptors (lithium, lamotrigine, dextromethorphan, methadone); autism spectrum disorder; developmental delay or intellectual disability; uncontrolled hypertension or hemodynamically significant cardiovascular disease (coronary artery disease, heart failure); pregnancy or breastfeeding; and allergy or prior adverse reaction to ketamine. All subjects continued their current medications throughout the trial.

Eligible patients were identified from the ED electronic tracking board and chart review. Research team members approached eligible patients and their families, explained the study, and obtained written consent from the parent/guardian and assent from the adolescent. If the parent/guardian was not present, he/she could provide verbal consent by phone and written consent electronically. Participants were then randomized to receive placebo or ketamine by the investigational drug pharmacist. The patient, family members, care team, and research team were blinded to group allocation.

### Measures

Depression and suicidality were measured using the BYI and SIQ (or SIQ–Junior, as appropriate). The BYI is a self-report scale to assess symptoms of depression, with five subscales: Depression Inventory; Anxiety Inventory (BAI-Y); Self-Concept Inventory (BSCI-Y); Anger Inventory; and Disruptive Inventory. Total scores range from 0–100 with scores > 69 associated with severe depression.^31^ A higher self-concept score is associated with lower risk of depression. The SIQ is a validated self-report measure to assess severity and frequency of suicidal thoughts. It consists of 30 items and is appropriate for students in grades 10–12. Scores range from 0–120, with scores > 41 associated with increased risk of suicide attempt in the following six months. The SIQ–JR consists of 15 items and is designed for students in grades 7–9. Scores range from 0–60, with scores > 31 associated with increased risk of suicide attempt in the following six months.^32^

These measures were selected because they were self-administered and did not require trained mental health professionals for completion. The BYI and SIQ were done at baseline and at 1 hour, 3 hours, 1 day, 3 days, and 7 days after treatment. Our primary outcome was the occurrence of reductions of ≥ 50% in both the BYI and SIQ/SIQ–JR at three hours after treatment. We recorded the occurrence of reductions of ≥ 50% in the scores at all other time points, as consistent with other studies on depression.^21^,^23^ To assess active suicidality, we also included a written yes/no question to the SIQ at all time points. If the subject was in the ED or our inpatient psychiatric facility, the questionnaires were completed in person. If the subject was transferred or discharged, contact was made via telephone by a member of the research team to complete the questionnaire. Participants were compensated with a $20 gift card after study completion.

### Study Procedures

The investigational drug services pharmacy provided either ketamine 0.2 mg/kg (minimum 10 mg, maximum 35 mg) or normal saline in a volume-matched syringe, based upon a predetermined randomization scheme known only to the pharmacy staff. We chose the ketamine dose based on the effectiveness of 0.2 mg/kg in adult ED patients.^25^–^28^ A sedation-credentialed emergency physician ordered the study drug (titled “investigational ketamine or placebo,” and a nurse administered the medication over two minutes with a study team member present. The nurse monitored subjects, recording heart rate, respiratory rate, blood pressure and oxyhemoglobin saturation at baseline and at five minutes and one hour after administration. Adverse events were monitored by the enrolling member of the study team (physician or medical student) and managed by the patient’s treating physician. After the one-hour monitoring period, the subjects resumed standard ED and psychiatric care. Standard care in our ED of suicidal patients included close monitoring by a behavioral health assistant outside the room with the patient door open and frequent reassessments by nursing. The patient’s nurse and emergency physician verified all home medications. There was also a social worker assessment every 12 hours and consultation with a psychiatrist for patients who were in the ED over 24 hours. Patients did not receive psychotherapy or initiate new psychotropic medications while in the ED.

### Sample Size

We calculated our sample size based on the precision of the measured effect size; 15 per group would allow us to demonstrate a difference in proportions achieving adequate response (≥ 50% reduction from baseline) of 0.6 between groups (12/15 in ketamine group versus 3/15 in placebo group), with a 95% confidence interval of 0.24–0.78.^21^

### Statistical Analyses

We summarized demographic characteristics of the study population using descriptive statistics. We conducted subanalyses of response rates and scores on high-severity subsets with baseline SIQ scores > 41 or SIQ–JR scores > 31 and baseline Beck Depression Inventory scores > 29. We omitted from analyses specific time points in which any subjects had missing values. We analyzed data using STATA 18 (StataCorp LLC, College Station, TX) and compared ketamine and placebo groups using the Fisher exact test for categorical data and the Wilcoxon rank-sum test for continuous data.

## RESULTS

During the study enrollment period from March 2022–December 2023, we screened 1,228 patients 12–17 years of age who presented to the ED with any behavioral chief complaint (CONSORT diagram, Figure 1). Of the 74 eligible patients, 16 were not approached due to patient sleeping or inadequate staffing, and 28 declined enrollment. The most common reasons for declining included parental concerns regarding insufficient data on ketamine in adolescents and adolescents’ refusal of IV placement. Of the 30 patients enrolled, 15 (50%) were randomized to the control group, and 15 (50%) were randomized to the intervention group. One participant in the placebo group did not meet the definition of treatment-resistant depression and was removed from analysis. Twelve subjects completed all study procedures and all scheduled questionnaires. The remainder had ≥ 1 missing BIY or SIQ score during the 7-day follow-up period after discharge. Of the enrolled subjects, 21 were transferred to inpatient psychiatric facilities, 6 were admitted to our crisis stabilization unit for 1–2 days, and 2 were discharged from the ED after crisis stabilization. There was no significant difference in disposition between the two groups. Median ED length of stay was two days.

Approximately two-thirds of enrollees were female, and one-fourth identified as non-binary (Table 1). This sex distribution was like that of all suicidal patients in the ED during the study period, which was 66% female. Approximately three-fourths of enrollees were White, and one-third were Hispanic. During the study period, about half of all suicidal patients in the ED were White and one-fourth were Hispanic. While all subjects had treatment-resistant depression, they often had comorbid psychiatric diagnoses, most commonly anxiety (66%). All subjects reported having trialed SSRIs. Subjects who received ketamine were significantly more likely to be currently taking aripiprazole (*P* = .03) and multiple medications (*P* = .05) than controls. There were no significant differences in median PHQ-9 scores, baseline BYI and SIQ scores between groups. Although not statistically significant, subjects who received ketamine were two times more likely to have a severe baseline PHQ-9 score than in the control group. There were no significant differences in medical comorbidities, which included type 1 diabetes mellitus, severe eczema, abnormal uterine bleeding, asthma, celiac disease, and migraines.

There were decreases from baseline in BDI-Y, BAI-Y, and SIQ scores and increases in BSCI-Y scores in both groups (Figures 2 and 3). However, none represented statistically significant differences between groups. Seven subjects of 15 in the ketamine group (47%) and four (29%) of the 14 in the placebo group had ≥ 50% reduction in score on at least one follow-up measurement. However, there was no statistically significant difference in the proportions with ≥ 50% reduction in scores (Table 2). The ketamine group included all four patients who experienced score reduction of ≥ 50% within three hours, which occurred in various combinations: 1 SIQ only (1); 1 BDI and BAI (1); BDI, BAI, and BSCI (1); and SIQ, BDI, and BAI (1). Of the seven subjects in the ketamine group with score reductions at any timepoints, four were within 1 hour, 1 within 1 day, and 2 within 3 days; 6 of 7 had sustained decrease at 7 days. Of the four subjects with score reductions in the placebo group, three were within 1 day and were sustained for 7 days, and 1 was at 7 days. Presence of active suicidality, elicited from 21 (72.4%) of subjects, did not significantly differ between groups at any time points.

Vital sign changes after administration of treatment differed little between groups, with only a significant difference in heart rate change in the ketamine group at five minutes compared to the placebo group (*P* = .004) (Table 3). All vital signs were normal at one hour. Self-reported adverse effects were more common in the ketamine group than the control group (*P* < .001) (Table 4). Nine (60%) in the ketamine group reported sensations compatible with ketamine’s known dissociative effect and reported feeling “weird” or “out of it” compared to one in the placebo group (7%, *P* = .004). Dizziness occurred in five participants (33%) in the ketamine group compared to none in the placebo group (*P* = .03). One subject in the ketamine group experienced a serious adverse event, consisting of altered mental status and abnormal jerking movements. She was treated with one dose of lorazepam by the clinical care team. There were no vital sign changes during this event, and the team documented a suspected psychogenic non-epileptic event. None of the patients required physical or chemical restraints while in the ED. No self-reported effects persisted beyond the one-hour observation period.

## DISCUSSION

This is the first study, to our knowledge, to examine the use of ketamine for depression and suicidal ideation in adolescents in the pediatric ED setting. There were decreases from baseline in BDI-Y, BAI-Y, and SIQ scores and increases in BSCI-Y scores in both groups (Figures 2 and 3). However, none represented statistically significant differences between groups. Patients who received a placebo also experienced improvement in scores, likely due to support from staff and follow-up psychiatric care.

Most patients did not experience serious adverse effects. The most common adverse effects reported by subjects receiving ketamine were consistent with mild dissociative symptoms. While dissociation may be psychologically distressing, especially if patients are unprepared, it may be important to ketamine’s effectiveness for depression. Luckenbaugh et al found that the dissociative effect of ketamine is correlated to its antidepressant properties.^33^ This may be due to ketamine’s disruption of the “default mode network,” a group of structures in the brain that controls one’s sense of self, ego, and repetitive thinking, and plays a role in depression. It is important for clinicians to be aware of this dissociative effect and to reassure patients that it is transient and expected.

Prior studies conducted in inpatient and outpatient psychiatric settings typically reported a dosing protocol with an infusion of 0.5 mg/kg IV given over 40 minutes.^9^–^13^ However, three studies in adult ED settings (two randomized controlled trials [RCT] and one open label) demonstrated that a single dose of ketamine 0.2 mg/kg IV given over 2–5 minutes provided rapid and transient relief of depressive symptoms and suicidality within hours.^25^–^27^ In the RCT by Domany et al, 88% of the ketamine group had resolved suicidal ideation within 90 minutes of infusion.^25^ We selected a low dose of ketamine at 0.2 mg/kg IV given over 2 minutes based on these studies and our institutional sedation protocol. Additionally, this dosing regimen was more feasible than a 40-minute infusion in the fast-paced ED setting.

Similar dosing was previously reported in an RCT in adolescents by Zhou et al, although repeated doses of 0.25 mg/kg were able to be used because the study took place in an inpatient setting.^22^ An RCT by Dwyer et al comparing 0.5 mg/kg IV ketamine to midazolam,^21^ and an open-label study by Cullen et al using 0.5 mg/kg IV ketamine both demonstrated reduction in depression scores in adolescents.^23^ While these studies and our study included patients with treatment-resistant depression who had already trialed at least 1–2 antidepressants, Lineham et al examined predictors of ketamine response in adolescents with depression and found that subjects who had fewer medication trials were more likely to respond to ketamine.^34^ Further studies are needed to determine the optimal dosing, frequency, and route of ketamine for adolescents, as well as to identify which patients are more likely to respond.

Another important aspect contributing to the effectiveness of ketamine and other psychedelics is the concept of “set and setting.” Set refers to the patient’s mindset and emotional state prior to treatment, and setting refers to the environment in which treatment occurs.^35^ Qualitative studies have found that set and setting were influential to the ketamine experience, and that a safe, calm, supportive environment is vital.^36^,^37^ Patients have reported feelings of awe, connectedness, altered perception of time and space, physical sensations, and ego dissolution. This may be distressing if patients are not properly counseled and prepared. While we did follow a script on counseling patients on expected effects, it is challenging to create a calm environment in an often chaotic ED. Studies on Emergency Psychiatric Assessment, Treatment, and Healing (EmPATH) units have shown that a designated calm, clinical space for patients experiencing behavioral health crisis can decrease boarding times and need for inpatient psychiatric hospitalization.^38^–^40^ Although creating such an environment within a traditionally chaotic ED can be challenging, future studies may evaluate pragmatic strategies such as noise-canceling headphones, music, or eye masks to reduce sensory overload, or assess ketamine administration within EmPATH units to determine whether environmental modification further improve treatment response and patient-centered outcomes.

## LIMITATIONS

The study had several limitations. Our sample size, based on an assumption of 50% reduction in scores, limited our ability to detect smaller differences. Larger trials powered to detect smaller effect sizes may reveal clinically important reductions in measures of depression and suicidal ideation. Because the study was conducted at a single site, a busy urban children’s hospital, our results may not be generalizable to all settings. There were more females than males enrolled, mirroring the gender distribution of suicidal patients seen in our ED. Due to the high volume of the ED, staffing limitations as well as bed availability created occasional challenges with enrollment. Members of the study team recruited patients when available, including during business hours and while on clinical shifts. Patients with behavioral health complaints often presented to the ED at night, and missed enrollments could have led to selection bias. However, many eligible patients remained in the ED and were available for recruitment in the morning. We also omitted scheduled study procedures when enrollees were sleeping during night shifts. Although missing data decreased the study’s power to detect differences, we prioritized patient comfort and avoided the confounding that could have resulted from the completion of study instruments by subjects awakened overnight.

We chose the most restrictive definition of treatment-resistant depression (trialed two anti-depressants) based on prior literature, which limited our eligible patients. However, this entry criterion was selected for patients with the highest severity of depression and who needed treatment most. Study instruments were only available in English, which excluded non-English speaking patients. We decided to use self-administered scales to facilitate data collection in the busy ED setting. Use of the SIQ was a major limitation, because it asked how subjects felt over the past 30 days, aiming to capture subjectivity of their sense of the last month rather than capturing how they felt at that moment. Thus, this scale may have been less useful for dynamic changes during the ED stay.

While we used validated instruments, they were not originally designed to capture hour-to-hour changes in mood and suicidal ideation. This may have limited the ability to detect short-term changes during the ED stay, and this should be considered when interpreting the results. Although the study was blinded, due to the self-reported physiologic effects of ketamine, both subjects and clinicians could have suspected which substance the subject had received. Subjects were followed for seven days, and some were lost to follow-up. Responses to BYI and SIQ at any time after the one hour monitored period may have been influenced by external factors other than the study agent, such as psychiatric care and hospital or home environment. Longer studies are needed to determine the duration of the effects of ketamine.

## CONCLUSION

Despite its effectiveness in the treatment of acute depression and suicidality in the adult ED setting, we found no significant differences in depression and suicidality scores between adolescent ED patients receiving a single dose of 0.2 mg/kg IV ketamine and those receiving placebo. Larger studies may reveal a clinically useful effect size and determine an optimal route and dosing for effectiveness of ketamine treatment in adolescents in the ED setting. If future research demonstrates its ability to lessen acute symptoms of adolescent depression and suicidality in the ED setting, ketamine could impact the adolescent mental health crisis by lowering the strain on EDs and inpatient psychiatry units.

## Figures and Tables

**Figure 1:**
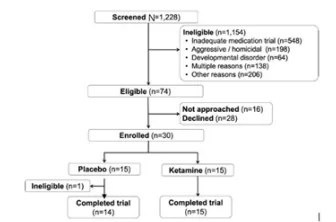
Consolidated Standards of Reporting Trials diagram depicting study enrollment for a randomized controlled trial comparing low-dose ketamine to placebo for adolescents presenting to the emergency department with suicidal ideation. This diagram outlines patient screening, enrollment, randomization, allocation, and follow-up.

**Figure 2:**
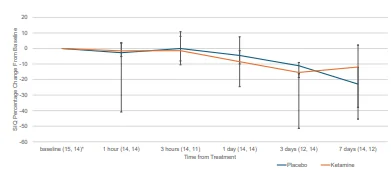
Percentage change in Suicidal Ideation Questionnaire (SIQ) scores among adolescents receiving low-dose ketamine vs placebo in the emergency department. The figure depicts mean percentage change from baseline SIQ scores at each assessment point, with error bars representing standard error (n = number of subjects). *SIQ*, Suicidal Ideation Questionnaire.

**Figure 3:**
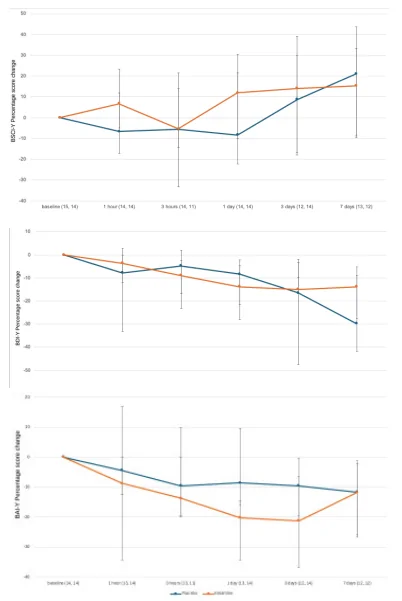
Percentage change in Beck Inventory Sub-Scores among adolescents receiving low-dose ketamine vs placebo in the emergency department. The figure depicts mean percentage change from baseline Beck Inventory scores at each assessment point, with error bars representing standard error. Top: Beck Self-Concept Inventory (BSCI-Y); Middle: Beck Depression Inventory (BDI-Y); Bottom: Beck Anxiety Inventory (BAI-Y).

**Table 1:** Baseline demographic and clinical characteristics of participants randomized to low-dose ketamine or placebo in a randomized controlled trial of adolescents presenting to the emergency department with suicidal ideation.

		Placebo group (n = 14)	Ketamine group (n = 15)	*P*
Gender Identity, n (%)				.13
Female		8 (57.1)	10 (66.7)	
Transgender male		1 (7.1)	0 (0)	
Nonbinary		5 (35.7)	2 (13.3)	
Male		0 (0)	3 (20.0)	
Age, yr., median (IQR)		15 (14, 16)	16 (15, 16)	.36
Ethnicity, Hispanic, n (%)				.24
Race, n (%)		6 (42.9)	3 (20.0)	1.00
White		11 (78.6)	12 (80.0)	
Asian		1 (7.1)	2 (13.3)	
Mixed		1 (7.1)	1 (6.7)	
Declined to answer		1 (7.1)	0 (0)	
Chronic health issues*		4 (28.6)	1 (6.7)	.17
Mental health diagnoses, n (%)				
Anxiety		10 (71.4)	9 (60.0)	.70
Other^1^		4 (28.6)	9 (60.0)	.14
ADHD		3 (21.4)	4 (26.7)	1.00
Eating disorder		2 (14.3)	6 (40.0)	.22
Trial medications, n (%)				
SSRI		14 (100)	15 (100)	.07
SNRI	Other	5 (35.7)	1 (6.7)	.02
Current medications, n (%)		1 (7.1)	8 (53.3)	
Aripiprazole		1 (7.1)	8 (53.3)	.02
Fluoxetine		3 (21.4)	4 (26.7)	1.00
Escitalopram		4 (28.6)	5 (33.3)	1.00
Multiple		2 (14.3)	8 (53.3)	.05
Other		7 (70.0)	6 (40.0)	.72
None		0 (0)	2 (13.3)	.48
Baseline scores, median (IQR)				
PHQ-9		18 (16–23)	22 (18–24)	.32.53
SIQ		120 (75–138)	129 (88–144)	.71
BSCI-Y		21.5 (19–25)	20 (13–30)	.43
BAI-Y		32 (30–41)	38 (30–43)	1.00
BDI-Y		42 (33–44)	40.5 (34–52)	.57
Disposition, n (%)				
Transferred to inpatient psychiatric facility				
Admitted crisis stabilization unit 1–2 days		11 (78.6)	10 (66.7)	
Discharged after crisis		3 (21.4)	3 (20.0)	
Admitted to crisis stabilization unit for 1–2 days		0	2 (13.3)	

* Placebo patients: 1 with severe eczema; 1 with abnormal uterine bleeding; 1 with asthma, celiac, endometriosis; and 1 with migraines. One ketamine patient had type 1 diabetes.

1 Other includes post-traumatic stress disorder (placebo group: 2, ketamine group: 3), obsessive-compulsive disorder (placebo group: 0, ketamine group: 2), personality disorder (placebo group: 1, ketamine group: 1), oppositional defiant disorder (placebo group: 1, ketamine group: 1).

*ADHD*, attention-deficit/hyperactivity disorder; *BAI-Y*, Beck Anxiety Inventory; *BDI-Y*, Beck Depression Inventory; *BSCI-Y*, Beck Self-Concept Inventory; *IQR*, interquartile range; *PHQ-9*, Patient Health Questionnaire-9; *SSRI*, selective serotonin reuptake inhibitor; *SNRI*, serotonin-norepinephrine reuptake inhibitor; *SIQ*, Suicidal Ideation Questionnaire; *yr*, years.

**Table 2:** Number of adolescents demonstrating a significant reduction in suicidal ideation after receiving low-dose ketamine vs placebo in the emergency department at various time points.

	Placebo group	Ketamine group	*P* value
Reduction by >			
50% in SIQ	0/14 (0)	2/14^1^ (14.3)	.48
1 hour	0/11^1^ (0)	2/14^1^ (14.3)	.49
3 hours	1/14 (7.1)	2/14^2^ (14.3)	1.00
1 day	2/14 (14.3)	3/13^2^ (23.1)	.65
3 days	2/12^2^ (16.7)	2/14^2^ (14.3)	1.00
7 days			
Reduction by >			
50% in BAI-Y	0/14 (0)	3/14^1^ (21.4)	.22
1 hour	0/11^1^ (0)	0/14^1^ (0)	-
3 hours	0/14 (0)	2/14^2^ (14.3)	.48
1 day	1/14 (7.1)	0/12^2^ (0)	1.00
3 days	1/12^2^ (8.3)	1/13^2^,^3^ (7.7)	1.00
7 days			
Reduction by >			
50% in BDI-Y	0/14 (0)	2/13^1^ (15.4)	.22
1 hour	0/11^1^ (0)	0/13^1^ (0)	-
3 hours	2/14 (14.3)	2/13^2^ (15.4)	1.00
1 day	1/14 (7.1)	3/12^2^ (25)	.31
3 days	1/12^2^ (8.3)	2/12^2^,^3^ (16.7)	1.00
7 days			
Active suicidality^4^			
Baseline	7/12 (63.7)	5/7 (71.4)	1.00
1 hour	6/12 (50.0)	2/8 (25.0)	.37
3 hours	1/9 (11.1)	2/7 (28.6)	.55
1 day	3/10 (30.0)	5/8 (62.5)	.34
3 days	2/12 (16.7)	1/6 (16.7)	1.00
7 days	1/12 (8.3)	1/7 (14.3)	1.00

Data are given as n/N (%).

1 Data missing for patients who were sleeping

2 Data missing for patients who were unable to be reached.

3 One patient completed the Suicidal Ideation Questionnaire but not the Beck Depression Inventory at day 7.

4 Data collected for 9 ketamine and 12 placebo patients.

*BAI-Y*, Beck Anxiety Inventory; *BDI-Y*, Beck Depression Inventory; *SIQ*, Suicidal Ideation Questionnaire.

**Table 3:** Median vital sign changes among adolescents receiving low-dose ketamine versus placebo in the emergency department.

	Placebo (median, IQR)	Ketamine (median, IQR)	*P* value
Baseline			
Heart Rate (bpm)	78 (72–92)	76.5 (65–83)	.35
Systolic blood pressure (mm Hg)	115 (104–123)	117 (110–126)	.45
Diastolic blood pressure (mm Hg)	66 (59–74)	64.5 (58–66)	.25
5-minute vitals change			
Heart Rate (bpm)	5 (−7, 0)	5 (−1, 8)	.004
Systolic blood pressure (mm Hg)	0.5 (−10, 6)	5.5 (1, 11)	.14
Diastolic blood pressure (mm Hg)	0 (−2, 4.5)	8 (0, 16)	.09
1-hour vitals change			
Heart Rate (bpm)	5.5 (−3, 7)	2 (1, 4)	.68
Systolic blood pressure (mm Hg)	−2.5 (−4, 7)	−2 (−5, 4)	.64
Diastolic blood pressure (mm Hg)	1.5 (−2, 8)	−1 (−7, 6)	.31

*bpm*, beats per minute; *IQR*, interquartile range.

**Table 4:** Self-reported adverse effects among suicidal adolescents receiving low-dose ketamine vs placebo in the emergency department.

	Placebo	Ketamine	*P* value
Adverse effects, n (%)			
None	10 (71.4)	1 (6.7)	< .001
Dissociation	1 (7.1)	9 (60.0)	.004
Drowsiness	2 (14.3)	4 (26.7)	.65
Dizziness	0 (0)	5 (33.3)	.03
Nausea/Vomiting	1 (7.1)	5 (33.3)	.10
Other^1^	1 (7.1)	6 (40.0)	.80

1 Other effects include nystagmus, visual hallucinations, headache, and anxiety.
